# The application of drug behavior management methods in the treatment of dental fear and oral diseases in children: A review

**DOI:** 10.1097/MD.0000000000037520

**Published:** 2024-03-22

**Authors:** Yuqi Huang, Chao Yang, Jingjing Nie, Manman Zeng, Huifang Kuang, Kaiyue Zheng, Honglan Sun, Xi Xie, Xiaoning He, Hai-Bin Luo, Wen Luo

**Affiliations:** a Department of Stomatology, First Affiliated Hospital of Hainan Medical University, Haikou, China; b School of Stomatology, Hainan Medical University, Haikou, China; c Department of Stomatology, The People’s Hospital of Longhua, Shenzhen, China; d Research and Development Department, Shenzhen Uni-medica Technology CO., Ltd, Shenzhen, China; e Department of Stomatology, The First Affiliated Hospital of Zhengzhou University, Zhengzhou, China; f Key Laboratory of Tropical Biological Resources of Ministry of Education and Hainan Engineering Research Center for Drug Screening and Evaluation, School of Pharmaceutical Sciences, Hainan University, Haikou, China.

**Keywords:** dental fear, drug behavior management, oral diseases, pediatric dentistry, sedation

## Abstract

Oral behavior management methods include basic behavior management methods and drug behavior management methods. In many cases, dental treatment that cannot be done simply through basic behavior management is not possible. The uncooperative behavior of children with dental fear in oral treatment has increased the demand for medication based behavior management methods. Drug sedation can provide more effective analgesic and anti-anxiety effects, thereby helping to provide comfortable, efficient, and high-quality dental services. This article will review the drug sedation methods selected in clinical treatment of pediatric dental fear in recent years, as well as the safety and effectiveness of commonly used drugs, in order to provide guidance for dental professionals in clinical practice.

## 1. Introduction

Pain, fear, and anxiety have long plagued children with oral diseases. Dentists face great challenges in treating children’s mouths, especially when children are separated from their parents, during local anesthesia, and even during simple noninvasive dental treatment.^[1]^ Most children show tension or physical resistance, and these non-cooperative behaviors are often clinically attributed to anxiety. Severe preoperative anxiety may increase the risk of postoperative complications, with potential long-term effects on psychological and developmental health in pediatric patients. The diurnal rhythm of sIgA secretion, which regulates anxiety, may be disrupted. Prolonged presence of anxiety symptoms, potentially due to a decrease in overall levels of sIgA, may carry the risk of compromised mucosal immunity.^[2,3]^ The negative psychological reaction of children in the diagnosis and treatment of oral diseases is called children’s dental fear. Such behavior is associated with increased sensitivity and reduced tolerance in children, and may have a serious impact on dental treatment, including delaying, terminating treatment, and reducing the quality of dental care provided.^[4]^ Epidemiological studies have shown that about 50% to 80% of children have dental fear.^[5]^

In recent years, the introduction of comfort-oriented induced therapy has made preoperative sedation more important. Due to the differences in children’s physical condition, cognitive ability and socio-emotional aspects, dentists need to take a series of behavioral measures to cater to individual needs and obtain parental cooperation. This method is called behavioral management in children’s oral treatment. Oral behavior management methods include basic behavior management and drug behavior methods. Children’s cooperation with dental surgery usually requires the entire dental team to adopt appropriate behavioral management strategies. For dental treatment that cannot be completed by basic behavior management, drug sedatives provide analgesic and anti-anxiety effects, enabling patients to respond appropriately to verbal commands and mild stimuli, thereby making dental treatment more friendly and effective for patients. This article summarizes the sedative methods commonly used in pediatric dentistry in recent years and the safety and efficacy of their representative drugs, so as to provide guidance for oral medical staff to deal with pediatric dental anxiety in clinical practice.

## 2. Grade classification of dental drug sedation in children

The latest version of sedation guidelines developed by the American Academy of Pediatrics and the American Academy of Pediatric Dentistry.^[6]^ According to the different degrees of inhibition of patient consciousness, the degree of sedation is divided into mild, moderate, deep sedation and general anesthesia (Table 1). The clinical manifestations of sedation vary greatly according to the type of drug, dose, method of administration, and the patient’s own situation.^[7]^ Under mild sedation, the children’s cognitive ability decreased, but they could still act autonomously and respond normally to verbal commands. The ventilation and cardiovascular function of the children were not affected. Moderate sedation, also known as “conscious sedation.” At this time, the child can perform the doctor’s verbal command with the help of tactile stimulation. In this state, the child can maintain natural ventilation, and cardiovascular function is usually not affected. Under deep sedation, children are difficult to wake up and can respond to repeated pain stimuli. At present, children’s ventilation function is impaired, autonomous ventilation may not be sufficient, assistance is needed to maintain airway patency, and cardiovascular function is usually not affected. Under general anesthesia, children lose consciousness, cannot wake up, have no response to pain stimulation, and need tracheal intubation assisted ventilation. At this time, children’s cardiovascular function may be damaged, often causing blood pressure to drop, and dynamic monitoring of respiratory and circulatory function is needed. To ensure the smooth progress of dental diagnosis and treatment in children with dental fear, the degree of drug sedation in children’s dental clinic often needs to reach moderate to deep sedation, while general anesthesia needs to be carried out in the operating room.

**Table 1:** Classification of sedation degree of dental drugs in children.

Degree	Ability		Function	
	Cognition	Autonomous behavior	Ventilation	Cardiovascular
Mild	Decrease	Presence	No effect	No effect
Moderate	Decrease	Decrease	Sustainability spontaneous ventilation	No effect
Deep	Severe decrease	Severe decrease	Insufficient spontaneous ventilation	No effect
General anesthesia	Loss	Loss	Tracheal intubation assisted ventilation	Broke

## 3. Management methods and commonly used drugs for pediatric dental drug behavior

### 3.1. Local anesthesia

Local anesthesia is mainly suitable for children who require localized surgery or specific treatments, such as tooth extraction and root canal therapy. It can effectively target the specific area, reduce pain sensation, and provide local sedation effect, while keeping the child awake. Local anesthetic drugs used in oral diagnosis and treatment can mainly be divided into esters and amides. However, due to the sensitization of ester compounds, they are gradually being abandoned in dental treatment. Amide drugs include lidocaine, articaine, procaine, mepivacaine, bupivacaine and more. Lidocaine is the gold standard for evaluating the safety and efficacy of other local anesthetic drugs, and is also the most commonly used local anesthetic in dentistry.^[8]^ In pediatric patients, clinical doctors must consider the dosage of lidocaine, which is a maximum dose of 4 mg/kg without the use of adrenaline. The pain experienced during anesthetic injection often arises from soft tissue injury caused by mucosal puncture, injection pressure, and excessive speed. Currently, computer-controlled local anesthetic delivery systems represent the state-of-the-art technology for achieving painless local anesthesia injections in dental practice.^[9,10]^ The reduction of anxiety and pain in pediatric patients is achieved through the administration of local anesthetics, while simultaneously controlling pressure and speed. The computer-controlled single tooth anesthesia system has undergone extensive validation in previous studies.^[11]^ The CALAJECT® system (Rønvig Dental MFG, Daugaard, Denmark) is an innovative painless anesthesia instrument recently introduced with 3 distinct modes: the first mode involves slow administration; the second mode combines 10 seconds of slow administration followed by rapid administration; and the third mode ensures faster and more effective supplementary anesthesia.^[12]^ The findings of a comparative study on the efficacy of single tooth anesthesia and CALAJECT in achieving painless anesthesia in pediatric patients suggest that both techniques warrant further investigation for their potential application in periodontal ligament injection.^[13]^ The continued effect of local anesthesia on soft tissue in children has also been a matter of particular concern, because even after the completion of dental treatment, the effect still exists in the form of tongue and lip numbness. In 13% of children, long-term local soft tissue anesthesia leads to soft tissue injury.^[14]^ In some cases, soft tissue injuries are misdiagnosed as infections, resulting in inappropriate hospitalization or use of antibiotics.^[15]^ Maegawa et al^[16]^ and Kubota’s^[17]^ research showed that 830 nm diode laser biological regulation increased blood flow by increasing vasodilation. In view of this mechanism of action, Annu et al^[18]^ used 810 nm and 660 nm photobiomodulation therapy in 20 children aged 4-8 years. The results showed that the local anesthesia reversal time of the 2 wavelengths was reduced by about 62 minutes on average compared with that without photobiomodulation therapy. However, the 660 nm wavelength was more effective in reversing the local anesthetic effect of soft tissue. For outpatient patients, it is recommended to use lidocaine with a shorter duration of action or ropivacaine and bupivacaine at the lowest concentration possible to avoid prolonged motor block (Table 2).

**Table 2:** Summary of the application and characteristics of children’s drug behavior management methods.

Methods Summary	Local anesthesia	Nitrous oxide inhalation	Oral drug sedation	Intravenous sedation	General anesthesia
Degree	Mild	Mild	Mild; moderate	Deep	General anesthesia
Advantage	Anesthesia target area	High safety; less complications	High acceptance; noninvasive	Sedation time and degree of good control	High comfort
Disadvantage	Short acting time	Need professional equipment; residual gases may be harmful	Unable to adjust the depth of sedation; first pass elimination	Difficult to establish venous access in the early stage	Long recovery time
Suitable population	Children with local treatment	Older (≥6 years) or cooperative children	Older (≥6 years) or cooperative children	Other approaches are not effective	Young (≤3 years) or uncooperative children
Example	Extraction of loose teeth; pulp canal therapy	Extraction of supernumerary teeth and impacted teeth; children with high blood pressure and heart disease; dizziness; needle fear	Preoperative diagnosis and therapeutic use before operation	Extraction of supernumerary teeth and impacted teeth; children with abnormal mental intelligence	Severe early childhood caries; surgery
Common drugs	Lidocaine	Mixed gas of nitrous oxide and oxygen	Midazolam	Propofol	Remazolam

### 3.2. Nitrous oxide inhalation sedation

Nitrous oxide inhalation sedation has become one of the widely accepted techniques worldwide. In the clinical work of European and American countries, 90% of pediatric dentists use nitrous oxide (N_2_O) inhalation for sedation and analgesia.^[19]^ This method is the preferred choice for achieving mild sedation in dental surgery, as it has a rapid onset and recovery time. The level of sedation can be adjusted by controlling the concentration of N_2_O. Its popularity has increased due to its long-term safety and minimal occurrence of complications.^[20]^ Researchers have explored various combinations of N_2_O and oxygen at different concentrations to induce sedative effects in pediatric surgeries associated with pain.^[21]^ Studies have shown that when the concentration of nitrous oxide is less than 50%, it can produce anti-anxiety and mild analgesic effects.^[22]^ At this time, patients can maintain normal respiratory, cardiovascular function and normal protective reflex. Liu et al^[23]^ found that the time to reach the maximum sedation level after inhalation of N_2_O was 5 to 6 minutes, which was effective in pediatric patients. The success rate was 93.6%, and the incidence of adverse reactions was 3.95%. The most common adverse reactions of inhalation sedation are dose-related nausea and vomiting.^[24]^ Inhalation of N_2_O and oxygen requires the cooperation of the child wearing a mask, so this method is suitable for older children who have good cooperation ability. It is not suitable for children with severe anxiety or respiratory system diseases. Considering the significant diffusion property of N_2_O, it should be avoided in patients who may have closed-cavity diseases such as intestinal obstruction, middle ear disease, and tension pneumothorax. In addition, the residual laughing gas in the clinic may endanger the reproductive system of medical staff (leading to abortion, infertility, etc.), inhibit the function of bone marrow and immune system, increase the incidence of cervical cancer, liver disease, etc., leading to mental disorders.^[25]^ Therefore, special attention should be paid to the selection of indications and the respiratory protection of medical staff to ensure good ventilation in the clinic and the tightness of the transmission pipeline.

### 3.3. Oral drug sedation

Oral drug sedation has a high acceptance rate among children and does not require complex equipment. It can achieve mild to moderate sedation without deep sedation, which is relatively safe. Oral sedatives are widely utilized as the primary method of pharmacological management during pediatric dental treatment.^[7]^ It is a noninvasive and convenient method of sedation that does not require injection or intubation. It is commonly used for older children, typically aged 6 and above, who are able to understand and follow the doctor’s instructions and have the ability to tolerate oral medications. However, the sedative effect of oral medications is greatly influenced by drug physicochemical factors and cannot be adjusted for sedation depth. Additionally, it has a longer onset of action compared to other administration routes and a relatively longer recovery time.^[26]^

Midazolam (MID) possesses sedative, anticonvulsant, and anxiolytic effects, rendering it one of the safest and most optimal drugs for pediatric patients. MID injection is an intravenous (IV) preparation that is poorly accepted by children due to its bitter taste.^[27,28]^ In clinical practice, its IV injection is often mixed with fruit juice or syrup to prepare an oral dosage form. This slightly sweet taste is more easily accepted by children, but it is currently less clinically used.^[29]^ MID administration can be performed 20 to 30 minutes before surgery, and the standard dose for moderate sedation in children with oral MID is usually 0.25 to 1 mg/kg. Drug efficacy can be achieved within 20 minutes, with a half-life of approximately 2.2 to 6.8 hours, significantly the drug effect does not seem to continue until 24 hours after surgery.^[30]^ A study found that oral administration of 0.5 and 0.75 mg/kg MID provides equally good sedative and anti-anxiety effects in children.^[31]^ Considering that the effectiveness and safety of MID are related to drug dosage, oral administration of 0.5 mg/kg MID solution is often chosen.^[32]^ Potential adverse effects of MID may include cognitive impairment, postoperative behavioral changes, and mild respiratory depression; however, these reactions generally do not impact ventilation or cardiovascular function significantly. These side effects are usually transient and mild in nature; therefore, close monitoring by medical personnel is advised during and after the administration of MID.^[33]^

### 3.4. IV sedation

IV sedation is the most rapid method for achieving anesthetic effects and offers optimal drug titration capabilities to attain specific blood concentrations. This method is suitable for pediatric patients who cannot receive N_2_O inhalation or have poor response to other routes of administration. The drug concentration can be strictly controlled through titration, ensuring a higher level of safety. However, children’s fear of needles can intensify anxiety, and their crying and struggling can increase the difficulty of venipuncture, requiring high technical sensitivity. IV sedation can induce a state of coma or deep sedation in children during the operation.

Propofol is a short-acting IV anesthetic with the advantages of fast onset, fast recovery, good stability, and prevention of nausea and vomiting. It is widely used for the induction and maintenance of IV anesthesia.^[34]^ The peak effect is usually achieved within 40 to 60 seconds after administration, with a duration of only 10 to 15 minutes, depending on the dosage and serum concentration of the drug.^[35]^ It inhibits sympathetic nerve activity and baroreceptor reflexes, leading to hemodynamic damage, which can lead to hypotension and bradycardia; It also increases the production of nitric oxide, leading to vasodilation.^[36]^ Propofol does not produce analgesic effects, therefore opioid or other drugs are needed to control pain. The addition of opioid anesthetics significantly reduced the dose required for propofol.^[37–39]^ Remifentanil is a synthetic, short-acting opioid used to induce and maintain sedation. It is used to induce and maintain sedation_._^[40]^ Whether propofol and remifentanil are sufficiently sedated mainly depends on whether the concentration of both in the brain or the effect compartment remains stable, and the dose should be compatible with the patient and the surgical procedure.^[41]^ To avoid causing vascular pain during the injection process, prevention can be achieved through slow injection and pre-application of lidocaine. For programmed sedation, the initial dose of propofol is slowly administered by IV injection at a dose of 0.5 to 1.0 mg/kg, and small doses of 0.5 mg/kg can be titrated every 3 to 5 minutes until the desired sedation level is achieved.^[42]^ As the serum concentration increases, the risk of adverse reactions such as respiratory arrest and bradycardia also increases. This rapid onset and short duration of action is ideal for shorter surgeries, and appropriate dosing intervals are necessary to avoid cardiopulmonary suppression.

### 3.5. General anesthesia

Dental general anesthesia is a management technique that uses anesthetic drugs to make children unconscious and undergo dental treatment under strict supervision.^[43]^ It can provide efficient oral diagnosis and treatment services for young children, not only providing a comfortable diagnosis and treatment experience for children, but also avoiding the inconvenience caused to parents by multiple follow-up visits during routine treatment.^[44]^ This method is commonly used for children under the age of 3 who are too young to cooperate with treatment, as well as for children with special needs such as intellectual disabilities and autism. The anesthesia method usually includes inhalation anesthesia and IV anesthesia. Tracheal intubation can be done through the nose or mouth, which is more convenient for dentists to operate. Local anesthesia can be added during tooth extraction, pulpotomy, and root canal treatment of pulpotomy teeth.

Remazolam is a water-soluble, ultra short acting IV benzodiazepine drug. Due to its excellent characteristics, including fast onset, organ independent metabolism, short duration, predictable recovery, availability of reversal drugs, hemodynamic stability, and no significant respiratory inhibition, Remazolam is expected to surpass other short-term sedatives currently in use.^[45]^ After its launch, Rimazolam was first used for sedation outside the operating room or non intubation general anesthesia, and later gradually applied for induction and maintenance of tracheal intubation general anesthesia. General anesthesia induction can be achieved through 2 methods: continuous IV injection of remidazolam and single IV injection. Continuous IV injection induction using Remazolam at a constant rate of 6 or 12mg/(kg·h). When the patient loses of consciousness (eye opening or speech response), the induction dose is stopped and switched to a maintenance dose. The patient experiences loses of consciousness after 97.2 seconds and 81.7 seconds of continuous injection at 2 different doses, respectively.^[46]^ Recently, in a pediatric case of glioma, stable myogenic motor evoked potential was recorded using 0.9 mg·kg^−1^·h^−1^ of remifentanil and 0.35 μg·kg^−1^·min^−1^ of remifentanil. It was found that the cardiovascular stability of remifentanil was better than that of propofol, and the propofol infusion syndrome was avoided.^[47]^ After surgery, there was no intraoperative memory or motor impairment. Horikoshi et al^[48]^ found that Remazolam could be safely used for general anesthesia in children with Du muscular dystrophy. Clinicians should be mindful of the potential risk of precipitation in the infusion tube and therefore avoid mixing Ringer’s Solution with remazolam as a maintenance solution. Remazolam, as a sedative and hypnotic component of balanced anesthesia, has excellent efficacy and safety, but there are also adverse reactions associated with remazolam during sedation and anesthesia. It has been reported that several patients experienced allergic reactions during the systemic induction of remazolam. Among them, 2 cases exhibited severe circulatory collapse.^[49,50]^ However, the incidence of allergic reactions in the general population using remazolam is still unclear and further research is needed to confirm. Due to the short clinical application time of remidazolam, no recommendation for IV administration of remidazolam has been proposed so far. At present, there is no comprehensive study to explore the effects of IV infusion of remidazolam on parameters such as spontaneous respiration and hemodynamics, and the effects of IV infusion of remidazolam on different ages are also unknown.

## 4. Conclusion and prospects

**Figure 1.**
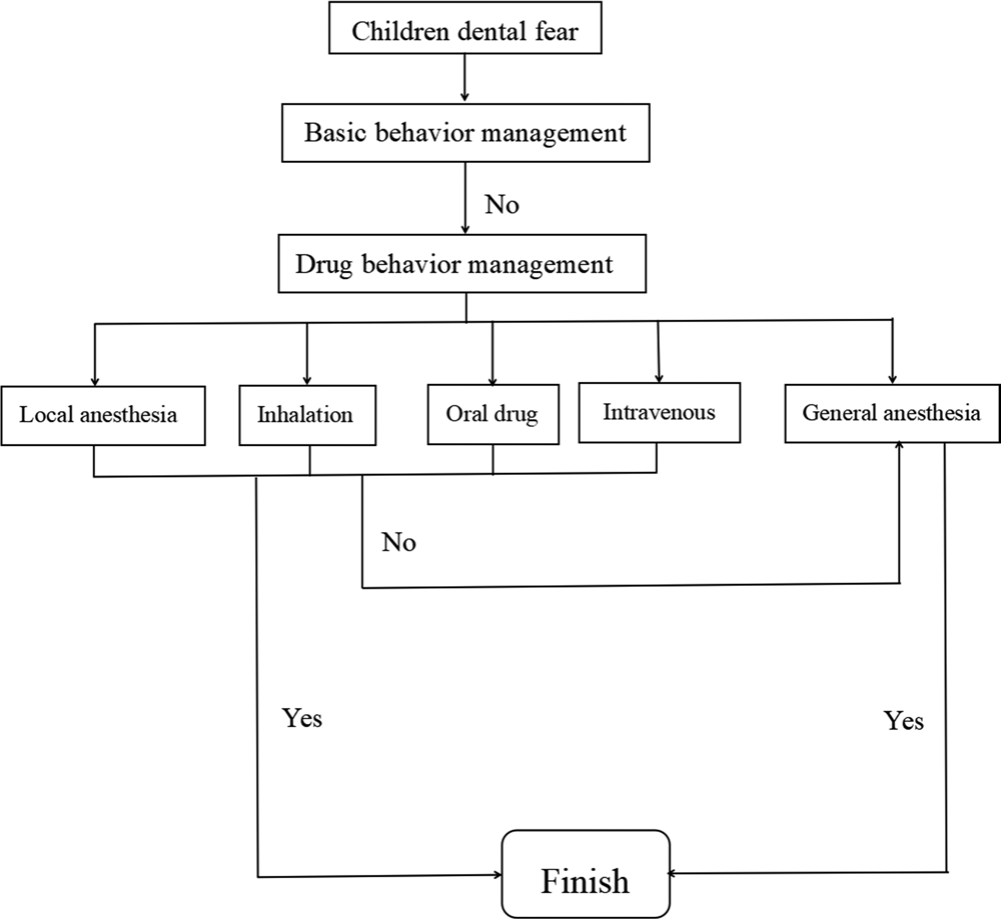
Dental fear behavior management flow chart of children.

Relieving anxiety and fear in uncooperative children and safely conducting dental treatments has always been a challenge for pediatric dentists. Failure to effectively manage and treat fear related to dental procedures may lead to lasting psychological effects or hinder future dental care and interventions. For dental treatments that cannot be completed through basic behavioral management, sedation or general anesthesia is often required, which can improve the child’s cooperation with future dental procedures (Fig. 1). During the pediatric sedation process, clinicians need to not only focus on the child’s psychological development and consider the impact on neurodevelopment, but also strictly adhere to the indications and contraindications of sedation techniques, sensitively identify the risk factors for preoperative anxiety in patients, and actively explore personalized intervention methods in order to provide a more comfortable and safe perioperative experience for the patients. Future research on pediatric dental sedation techniques mainly focuses on summarizing and refining the guidelines for the proper use of commonly used sedative drugs in pediatric dentistry, exploring new sedative drugs and appropriate combination therapies to improve the overall quality of pediatric oral diagnosis and treatment.

## Author contributions

**Conceptualization:** Wen Luo, Hai-Bin Luo, Xiaoning He.

**Data curation:** Manman Zeng, Huifang Kuang, Kaiyue Zheng.

**Formal analysis:** Honglan Sun.

**Investigation:** Kaiyue Zheng, Huifang Kuang, Xi Xie.

**Writing – original draft:** Yuqi Huang, Chao Yang, Jingjing Nie.

**Writing – review and editing:** Wen Luo, Hai-Bin Luo, Xiaoning He.
